# Bile Biochemistry Following Liver Reperfusion in the Recipient and Its Association With Cholangiopathy

**DOI:** 10.1002/lt.25738

**Published:** 2020-07-21

**Authors:** Rohit Gaurav, Niroshan Atulugama, Lisa Swift, Andrew J. Butler, Sara Upponi, Rebecca Brais, Michael Allison, Christopher J. E. Watson

**Affiliations:** ^1^ Cambridge Transplant Unit Addenbrooke’s Hospital Cambridge University Hospitals National Health Service Trust Cambridge United Kingdom; ^2^ Department of Radiology Addenbrooke’s Hospital Cambridge University Hospitals National Health Service Trust Cambridge United Kingdom; ^3^ Department of Pathology Addenbrooke’s Hospital Cambridge University Hospitals National Health Service Trust Cambridge United Kingdom; ^4^ Department of Medicine Addenbrooke’s Hospital Cambridge University Hospitals National Health Service Trust Cambridge United Kingdom; ^5^ Department of Surgery Addenbrooke’s Hospital Cambridge University Hospitals National Health Service Trust Cambridge United Kingdom; ^6^ National Institute for Health Research Cambridge Biomedical Research Centre and the National Institute for Health Research Blood and Transplant Research in Organ Donation and Transplantation National Health Service Blood and Transplant at University of Cambridge and Newcastle University Cambridge United Kingdom

## Abstract

Cholangiocytes secrete bicarbonate and absorb glucose, producing bile with alkaline pH and low glucose content. These functions of cholangiocytes have been suggested as a marker of bile duct viability during normothermic ex situ liver perfusion, and they are now monitored routinely after reperfusion in our center. In this study, we reviewed the composition of bile immediately after reperfusion in liver transplant recipients to determine normal posttransplant parameters and the predictive value of bile biochemistry for the later development of cholangiopathy. After reperfusion of the liver graft, a cannula was placed in the bile duct to collect bile over a median 44‐minute time period. The bile produced was analyzed using a point‐of‐care blood gas analyzer (Cobas b221, Roche Diagnostics, Indianapolis, IN). A total of 100 liver transplants (35 from donation after circulatory death and 65 from donation after brain death) were studied. Median bile pH was 7.82 (interquartile range [IQR], 7.67‐7.98); median bile glucose was 2.1 (1.4‐3.7) mmol/L; median blood‐bile‐blood pH difference was 0.50 (0.37‐0.62); and median blood‐bile glucose difference was 7.1 (5.6‐9.1) mmol/L. There were 12 recipients who developed cholangiopathy over a median follow‐up of 15 months (IQR, 11‐20 months). Bile sodium (142 versus 147 mmol/L; *P* = 0.02) and blood‐bile glucose concentration differences (5.2 versus 7.6 mmol/L; *P* = 0.001) were significantly lower and were associated with ischemic cholangiopathy. In conclusion, bile biochemistry may provide useful insights into cholangiocyte function and, hence, bile duct viability. Our results suggest bile glucose is the most sensitive predictor of cholangiopathy.

**Abbreviations**
ALD: alcohol‐related liver disease
ALF: acute liver failure
AUC: area under the curve
BMI: body mass index
CIT: cold ischemia time
DBD: donation after brain death
DCD: donation after circulatory death
DLI: United Kingdom donor liver index
ERCP: endoscopic retrograde cholangiopancreatography
HAT: hepatic artery thrombosis
HCV: hepatitis C virus
HbO_2_: hemoglobin oxygen
IC: ischemic cholangiopathy
IQR: interquartile range
MRCP: magnetic resonance cholangiopancreatography
MRI: magnetic resonance imaging
NAFLD: nonalcoholic fatty liver disease
NESLiP: normothermic ex situ liver perfusion
NRP: normothermic regional perfusion
PBC: primary biliary cholangitis
PSC: primary sclerosing cholangitis
pCO_2_: partial pressure of carbon dioxide
pO_2_: oxygen
ROC: receiver operating characteristic
SD: standard deviation
UKELD: United Kingdom Model for End‐Stage Liver Disease
UW: University of Wisconsin
WIT: warm ischemia time

The availability of novel ex situ normothermic liver perfusion techniques affords the opportunity to assess livers prior to transplantation^(^ ^1^, ^2^, ^3^, ^4^ ^)^ and to provide reassurance as to their viability.^(^ ^5^ ^)^ This is particularly so for livers from donation after circulatory death (DCD) donors, where primary nonfunction and posttransplant cholangiopathy are common. Although measures of hepatocyte damage and viability, such as transaminase release and lactate metabolism, are well recognized and accepted in clinical liver transplantation, less clear are the best indicators of bile duct viability. We have previously hypothesized that biliary pH and glucose are possible candidates for predicting posttransplant cholangiopathy in livers undergoing ex situ normothermic perfusion because they reflect cholangiocyte function.^(^ ^4^, ^5^ ^)^

Bile is produced by hepatocytes and undergoes modification as a result of absorptive and secretory processes by cholangiocytes lining the bile canaliculi and higher‐order ducts.^(^ ^6^, ^7^ ^)^ Transmembrane channels in these cells secrete bicarbonate and reabsorb glucose, producing bile with alkaline pH and low glucose content.^(^ ^8^, ^9^ ^)^ Deprotonation of glycine‐conjugated bile salts renders them less polar and less able to cross cell membranes in an uncontrolled manner, effectively deactivating them during their passage through the smaller bile ducts in the biliary tree.^(^ ^9^, ^10^ ^)^ Damage to the cholangiocytes during ischemia is thought to be associated with the development of intrahepatic biliary strictures.

Following on from our observations on livers perfused ex situ, we introduced routine testing of the biochemistry of the bile produced soon after reperfusion in liver recipients. We hypothesized that the biochemistry of bile at this time point may also provide information regarding the likelihood of future cholangiopathy. The aim of this article is to review the biochemical data we have collected and to see whether the same parameters believed to be important in predicting cholangiopathy during ex situ perfusion applied to reperfusion in vivo.

## Patients and Methods

### Bile Collection and Analysis

The study was conducted as a “service evaluation” of our practice of routine bile chemistry analysis. We studied deceased donor livers transplanted at our center between March 2017 and October 2018 for which the data were available for bile analysis. The donor bile duct was flushed with University of Wisconsin (UW) solution at the time of organ retrieval as a standard practice. Following reperfusion of the liver graft, removal of the donor gallbladder, and satisfactory rounds of hemostasis, a 5‐Fr infant feeding tube was introduced into the donor bile duct and secured with a purse‐string suture. Bile flow was diverted into an open container placed at the level of the operating table. A hemostatic break in surgery of approximately 30‐45 minutes was routinely taken after arterial reperfusion and donor cholecystectomy. A sample of the bile produced during this period was assessed using a Cobas b 221 (Roche Diagnostics, Indianapolis, IN) point‐of‐care blood gas analyzer. Directly measured parameters from this system include pH, partial pressure of carbon dioxide (pCO_2_), oxygen (pO_2_), and hemoglobin oxygen (HbO_2_) saturation, together with concentrations of sodium, potassium, chloride, calcium, glucose, and lactate. Bicarbonate and hydrogen ions are derived parameters in the analyzer, and the analyzer is only able to measure pH in a range between 6 and 8. Values higher than 8 were read as high and out of range. We have included these out‐of‐range values as 8 for data calculation purposes.

Patients were excluded from the study whenever the bile sample was not assessed or was not available for assessment after reperfusion. The reasons ranged from there not being a surgical break before bile duct reconstruction, to technical problems in tube placement with leakage, or there not being enough bile production for assessment.

### Clinical Data

Contemporaneously recorded clinical data were obtained from the electronic patient record (Hyperspace 2014 IU 1; Epic Systems Corporation, Madison, WI). The study was undertaken as a service evaluation of the utility of a bile chemistry analysis after reperfusion. Operative notes were assessed for the type of graft (donation after brain death [DBD] or DCD); the use of normothermic ex situ liver perfusion (NESLiP) or in situ normothermic regional perfusion (NRP); and anastomotic warm ischemia time (WIT) and cold ischemia time (CIT) as recorded during transplantation. Anastomotic WIT was defined as the time between the allograft being removed from ice and the restoration of blood flow by releasing the vascular clamps in the recipient. All but 1 of the livers were reperfused on the portal vein first. CIT was defined as the time interval between cold in situ flush with UW preservation solution and reperfusion in the recipient or as the start of ex situ normothermic machine perfusion, if that was used. The time to portal vein revascularization and hepatic artery clamp release were compared to define any influence of portal and hepatic arterial flows on the development of cholangiopathy.

The distal end of the donor bile duct was trimmed routinely before bile duct anastomosis. This trimmed end was fixed in formalin and sent for histological assessment of injury by dedicated liver pathologists who were unaware of the bile biochemistry findings. Damage was graded based on stromal necrosis (none = grade 0; ≤25% necrosis = mild, grade 1; >25%‐50% necrosis = moderate, grade 2; and >50% necrosis = severe, grade 3). The score was dichotomized into livers with low bile duct injury scores (none and mild injury) and livers with high bile duct injury scores (moderate and severe injury) for comparison.

All posttransplantation data were collected during routine clinical follow‐up of the recipients. Those who had persistent rise in alkaline phosphatase on follow‐up or as clinically indicated underwent magnetic resonance cholangiopancreatography (MRCP). Examinations were performed on a 1.5T magnetic resonancy system (GE Medical Systems, Milwaukee, WI), using a standard MRCP protocol. Imaging features of ischemic cholangiopathy (IC) were graded subjectively (modified from a study by Buis et al.^(^ ^11^ ^)^) as mild, moderate, and severe on the basis of the extent and number of strictures, degree of narrowing, biliary dilatation, and the presence of casts or calculi. Arterial stenosis or thromboses were excluded by imaging.

### Statistical Analysis

The follow‐up analysis of the study population ended in June 2019. Discrete variables were reported as absolute number and percentage. Continuous variables are presented as mean and standard deviation (SD) or as median and interquartile range (IQR) when data were nonparametric. Comparisons between groups were performed using Fisher’s exact test for categorical variables and Mann‐Whitney U test or Kruskal‐Wallis test (with post hoc Dunn’s test) for independent continuous variables. In addition, the predictive value of bile biochemistry to predict cholangiopathy was assessed by calculating the area under the receiver operating characteristic (AUROC) curve. The statistical level of significance was set at *P* < 0.05. All statistical analyses were performed using SPSS statistics software, version 21.0 for Windows (IBM, Chicago, IL) and Prism, version 8.0.0 for Windows (GraphPad Software, La Jolla, CA).

## Results

### Donor and Recipient Characteristics

A total of 202 liver transplants were performed between March 2017 and October 2018. Postreperfusion bile biochemistry data were available for 100 (49.5%) recipients, 35 from DCD and 65 from DBD donors. Out of 35 DCD liver grafts, 13 (37.1%) were recovered using NRP, and 18 livers (8 DBDs and 10 DCDs) with high United Kingdom donor liver index (DLI) were subjected to NESLiP once they reached our hospital. Only 1 liver underwent both interventions (Table 1). Median follow‐up of the study is 15 months (IQR, 11‐20 months).

**Table 1:** Donor and Graft Characteristics

	Overall (n = 100)	Graft Type			Presence of Cholangiopathy		
		DBD (n = 65)	DCD (n = 35)	*P* Value	No Cholangiopathy (n = 88)	Cholangiopathy (n = 12)	*P* Value
Age, years	47.7 ± 17	49.5 ± 17.3	44.3 ± 16	0.12	47 ± 17.5	53.4 ± 10.6	0.21
Sex, male	49 (49.0)	32 (49.2)	17 (48.6)	0.90	40 (45.5)	9 (75.0)	0.07
BMI, kg/m^2^	26.7 ± 4.8	26.7 ± 4.6	26.8 ± 5.4	0.76	26.8 ± 5	26.5 ± 3.7	0.88
DLI	1.3 (1.0‐1.6)	1.1 (0.9‐1.3)	1.9 (1.5‐2.2)	0.001	1.2 (1.0‐1.5)	1.8 (1.3‐2.1)	0.001
ABO type							
Identical	94	63 (96.9)	31 (88.6)	0.18	83 (94.3)	11 (91.7)	0.55
Compatible	6	2 (3.1)	4 (11.4)		5 (5.7)	1 (8.3)	
Machine perfusion							
None	68	57 (87.7)	11 (31.4)		59 (67.0)	9 (75.0)	
NRP	13	0 (0.0)	13 (37.1)	0.001	13 (14.8)	0 (0.0)	0.50
NESLiP	18	8 (12.3)	10 (28.6)		15 (17.0)	3 (25.0)	
NRP plus NESLiP	1	0 (0.0)	1 (2.9)		1 (1.1)	0 (0.0)	
Asystolic WIT, minutes	11 (10‐15)	—	11 (10‐15)	—	12 (10‐15)	11 (9‐15)	0.59
CIT, minutes	468 (378‐637)	516 (395‐663)	406 (328‐500)	0.01	473 (376‐638)	456 (388‐508)	0.99
Anastomotic WIT, minutes	38 (33‐45)	38 (33‐48)	36 (32‐42)	0.29	38 (33‐49)	37 (30‐39)	0.17
Arterial reperfusion time, minutes	78 (70‐94)	78 (71‐97)	80 (65‐92)	0.33	80 (70‐97)	74 (67‐92)	0.35

NOTE:

Data are given as n (%), mean ± SD, or median (IQR).

The donor and recipient characteristics were similar when compared with the type of donor graft and the development of cholangiopathy (Tables 1 and 2). The overall median DLI of the grafts was 1.3 (1.0‐1.6) with DCD grafts having higher DLI than DBD grafts (1.9 versus 1.1; *P* = 0.001). Similarly, the DLI was higher for grafts that subsequently developed cholangiopathy (1.8 versus 1.2; *P* = 0.001), but in part, this may reflect the higher weighting the DLI gives to DCD grafts. Median (IQR) CIT was 468 minutes (378‐637) and anastomotic WIT was 38 minutes (33‐45 minutes) with no difference in the timing between the groups based on development of cholangiopathy. However, median CIT was significantly longer for DBD grafts compared with DCD grafts (516 versus 406 minutes; *P* = 0.01).

**Table 2:** Recipient Demographics

	Overall (n = 100)	Graft Type			Presence of Cholangiopathy		
		DBD (n = 65)	DCD (n = 35)	*P* Value	No Cholangiopathy (n = 88)	Cholangiopathy (n = 12)	*P* Value
Age, years	55.7 ± 11	55.7 ± 11.4	55.9 ± 10.4	0.98	55.6 ± 11	56.4 ± 10	0.82
Sex, male	64 (64.0)	38 (58.5)	26 (74.3)	0.09	53 (60.2)	11 (91.7)	0.05
BMI, kg/m^2^	27.8 ± 5.6	27.3 ± 5.6	28.6 ± 5.5	0.18	27.8 ± 5.8	27.6 ± 4.2	0.92
UKELD	54 (52‐58)	55 (52‐58)	54 (52‐57)	0.12	54 (52‐58)	53 (51‐55)	0.1
Etiology							
ALD	23 (23.0)	15 (23.1)	8 (22.9)		22 (25.0)	1 (8.3)	
NAFLD	26 (26.0)	13 (20.0)	13 (37.1)		25 (28.4)	1 (8.3)	
PSC	16 (16.0)	12 (18.5)	4 (11.4)		12 (13.6)	4 (33.3)	
HCV	12 (12.0)	6 (9.2)	6 (17.1)		9 (10.2)	3 (25.0)	
PBC	5 (5.0)	4 (6.2)	1 (2.9)	—	5 (5.7)	0 (0.0)	—
ALF	2 (2.0)	2 (3.1)	0 (0.0)		2 (2.3)	0 (0.0)	
HAT	2 (2.0)	2 (3.1)	0 (0.0)		1 (1.1)	1 (8.3)	
Others	14 (14.0)	11 (16.9)	3 (8.6)		12 (13.6)	2 (16.7)	
Malignancy	20 (20.0)	9 (13.8)	11 (31.4)	0.07	14 (15.9)	6 (50)	0.002
Retransplantation	6 (6.0)	6 (9.2)	0 (0.0)	—	5 (5.7)	1 (8.3)	0.55
IC	12 (12.0)	4 (6.2)	8 (22.9)	0.02	—	—	—

NOTE:

Data are given as n (%), mean ± SD, or median (IQR).

Nonalcoholic fatty liver disease (NAFLD) formed the most common etiology (26%) of liver failure, followed by alcohol‐related liver disease (ALD; 23%); hepatocellular carcinoma was present in 19 recipients. There were 6 recipients who had previous liver transplants and received a second graft. An unexpected cholangiocarcinoma was identified in 1 of the explanted livers and that patient subsequently died from recurrence. A total of 12 (12%) patients developed IC, two‐thirds of whom were DCD liver recipients (DCD, n = 8, 22.8%; DBD, n = 4, 6.2%; *P* = 0.02) and half (n = 6) of those developing IC underwent transplantation for malignancy (*P* = 0.002). No liver from a donor that underwent NRP developed cholangiopathy.

### Bile Gas Parameters

Bile was collected over a median (IQR) break time of 44 minutes (37‐50 minutes). Median bile pH was 7.82 (7.67‐7.98), and median glucose was 2.1 mmol/L (1.4‐3.7 mmol/L) (Table 3). Table 4 shows the postreperfusion bile biochemistry of various grafts based on donor type and preservation technique. The median difference between bile and blood pH values was 0.50 (0.37‐0.62), whereas the median difference between blood and bile glucose was 7.1 (5.6‐9.1) mmol/L. Bicarbonate, being a derived parameter, was not recordable in 22 recipients whose bile pH was higher than 8. Of the known values, median bile bicarbonate was 23.1 (19.3‐27.1). Bile pH and glucose difference in standard DBD livers were similar to DCD livers undergoing NRP; the lowest bile pH (7.31) and highest glucose (11.7 mmol/L) were seen in standard DBD donor livers. The standard DCD livers had significantly low blood‐bile glucose difference as compared with standard DBD livers (4.8 versus 8.6 mmol/L; *P* = 0.002).

**Table 3:** Postreperfusion Bile Biochemistry

Parameters	n	Median	IQR	Minimum	Maximum
pH	100	7.82	7.67‐7.98	7.31	>8
pCO_2_, kPa	99	2.0	1.1‐3.0	0.6	5.5
${\text{HCO}}_{3}^{-}$, mmol/L*	78	23.1	19.3‐27.1	12.5	40.5
Na^+^, mmol/L	96	146	143‐149	94	159
K^+^, mmol/L	94	5.0	4.5‐5.6	3.4	8.7
Cl^–^, mmol/L^†^	40	110	106‐115	82	148
Glucose, mmol/L	97	2.1	1.4‐3.7	0.6	11.7
Lactate, mmol/L	98	2.0	1.3‐2.8	0.5	8.3

* The ${\text{HCO}}_{3}^{-}$ value was not available in 22 patients where the pH was out of range because it is a derived parameter.

^†^ The chloride module was added to the analyzer midway through the study.

**Table 4:** Bile Biochemistry According to the Preservation Technique of the Graft

	DBD (n = 57)	DCD (n = 11)	NRP (n = 13)	NESLiP (n = 18)*	*P* Value
pH	7.82 (7.67‐7.98)	7.79 (7.54‐7.94)	7.82 (7.65‐8.0)	7.87 (7.67‐8.0)	0.67
Glucose, mmol/L	2.5 (1.6‐4.1)	2.6 (2.2‐8.7)	1.2 (0.9‐2.2)	1.7 (1.4‐2.4)	0.005^†^
pCO_2_, kPa	2.1 (1.1‐3.1)	2.2 (1.6‐3.5)	2.0 (1.2‐4.0)	1.6 (1.0‐2.9)	0.37
Na^+^, mmol/L	146 (143‐150)	143 (139‐148)	146 (143‐149)	148 (142‐149)	0.38
K^+^, mmol/L	5.1 (4.8‐5.6)	5.2 (4.1‐5.5)	4.7 (4.3‐5.2)	4.9 (4.4‐5.8)	0.56
${\text{HCO}}_{3}^{-}$, mmol/L	22.5 (19.3‐26.8)	22.5 (19.6‐24.2)	29.1 (18.5‐35.6)	23.1 (16.2‐27.2)	0.48
Bile‐blood pH difference	0.49 (0.37‐0.62)	0.39 (0.20‐0.51)	0.53 (0.40‐0.62)	0.53 (0.40‐0.67)	0.32
Blood‐bile glucose difference, mmol/L	8.6 (6.2‐10.4)	4.8 (3.5‐6.5)	6.2 (5.9‐8.0)	6.6 (4.8‐8.4)	0.001^‡^
Bile‐blood Na difference, mmol/L	8 (5‐12)	5 (2‐11)	7 (6‐11)	7 (5‐11)	0.38

NOTE:

Data are given as median (IQR). One liver had both NRP and NESLiP and was excluded.

* NESLiP group includes 8 DBD livers and 10 DCD livers.

^†^ DBD versus NRP, *P* = 0.03; DCD versus NRP, *P* = 0.015 (Dunn’s pairwise test with Bonferroni correction).

^‡^ DBD versus DCD, *P* = 0.002.

### DBD and DCD

Bile biochemistry with regard to DBD and DCD livers was similar for all parameters except for the difference between the concentration of blood and bile glucose (Table 5). The median glucose difference was 8.4 mmol/L (6.1‐10.4 mmol/L) in DBD grafts as compared with 6.2 mmol/L (3.8‐7.3 mmol/L) in DCD grafts (*P* = 0.001).

**Table 5:** Biochemistry According to the Type of Graft and Presence of Cholangiopathy

Parameters	Bile						Blood
	Graft Type			Presence of Cholangiopathy			
	DBD (n = 65)	DCD (n = 35)	*P* Value	No Cholangiopathy (n = 88)	Cholangiopathy (n = 12)	*P* Value	
pH	7.85 (7.67‐7.98)	7.80 (7.67‐7.96)	0.97	7.86 (7.67‐7.98)	7.74 (7.53‐7.86)	0.12	7.31 (7.28‐7.35)
Glucose, mmol/L	2.1 (1.5‐3.8)	1.8 (1.2‐3.5)	0.31	1.9 (1.4‐3.7)	2.5 (1.8‐5.7)	0.23	9.8 (7.8‐12.6)
pCO_2_, kPa	2.0 (1.1‐3.0)	2.0 (1.2‐3.0)	0.97	1.9 (1.1‐3.0)	2.3 (1.4‐3.9)	0.27	5.2 (4.8‐5.6)
Na^+^, mmol/L	146 (143‐150)	146 (143‐149)	0.68	147 (143‐150)	142 (139‐148)	0.02	138 (136‐141)
K^+^, mmol/L	5.0 (4.8‐5.8)	4.9 (4.3‐5.4)	0.12	5.0 (4.5‐5.5)	4.9 (4.1‐5.9)	0.74	4.2 (3.8‐4.5)
Cl^−^, mmol/L	110 (105‐113)	109 (106‐125)	0.33	109 (106‐114)	113 (104‐129)	0.47	104 (102‐105)
${\text{HCO}}_{3}^{-}$, mmol/L	22.5 (19.1‐27.3)	23.2 (19.5‐26.6)	0.59	23.2 (19.3‐27.4)	21.8 (19.4‐24.2)	0.29	19.5 (18‐20.6)
Lactate, mmol/L	2.1 (1.4‐3.1)	1.9 (1.0‐2.5)	0.13	2.1 (1.3‐2.8)	1.6 (1.4‐2.6)	0.56	2.1 (1.6‐3.1)
Bile‐blood pH difference	0.49 (0.37‐0.63)	0.51 (0.35‐0.61)	0.76	0.51 (0.37‐0.62)	0.40 (0.20‐0.62)	0.19	—
Blood‐bile glucose difference, mmol/L	8.4 (6.1‐10.4)	6.2 (3.8‐7.3)	0.001	7.6 (5.9‐9.3)	5.2 (3.1‐6.5)	0.001	—
Bile‐blood Na difference, mmol/L	7.4 (5.3‐11.0)	6.6 (4.8‐11.0)	0.46	7.7 (5.7‐11.0)	3.4 (1.2‐6.5)	0.01	—

NOTE:

Data are given as median (IQR).

### Cholangiopathy

**Fig. 1:**
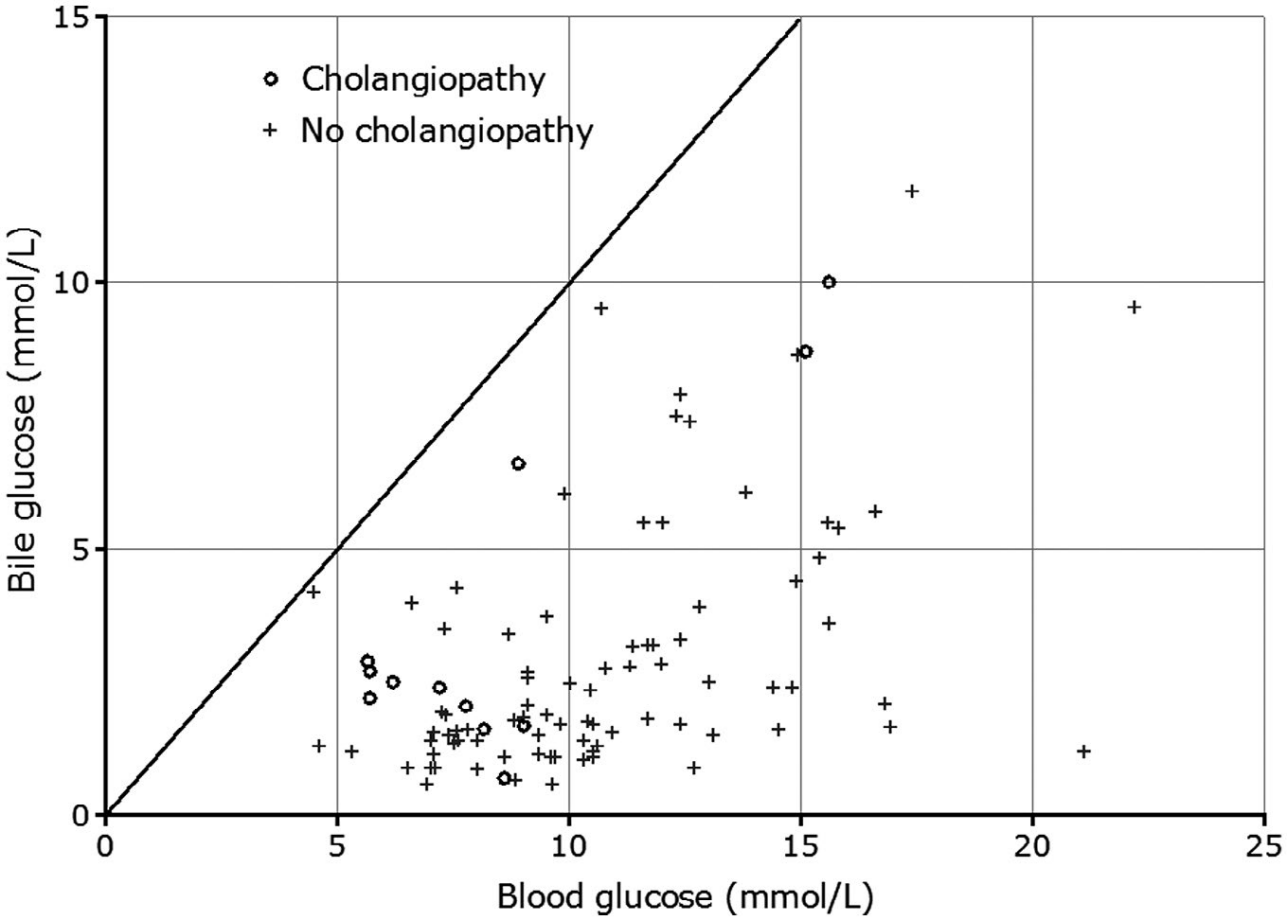
A graph illustrating the relationship of glucose concentration in the bile and blood after reperfusion of the liver in the recipients. The straight line indicates where bile and blood glucose are the same. We would expect all readings to lie below the line. Patients with cholangiopathy (indicated by the circles) had less difference between biliary and blood glucose, but there were patients without cholangiopathy whose values were on or near the line of equivalence. The graph also illustrates the saturation of the glucose transporter, where the bile glucose starts at a higher blood glucose concentration.

**Fig. 2:**
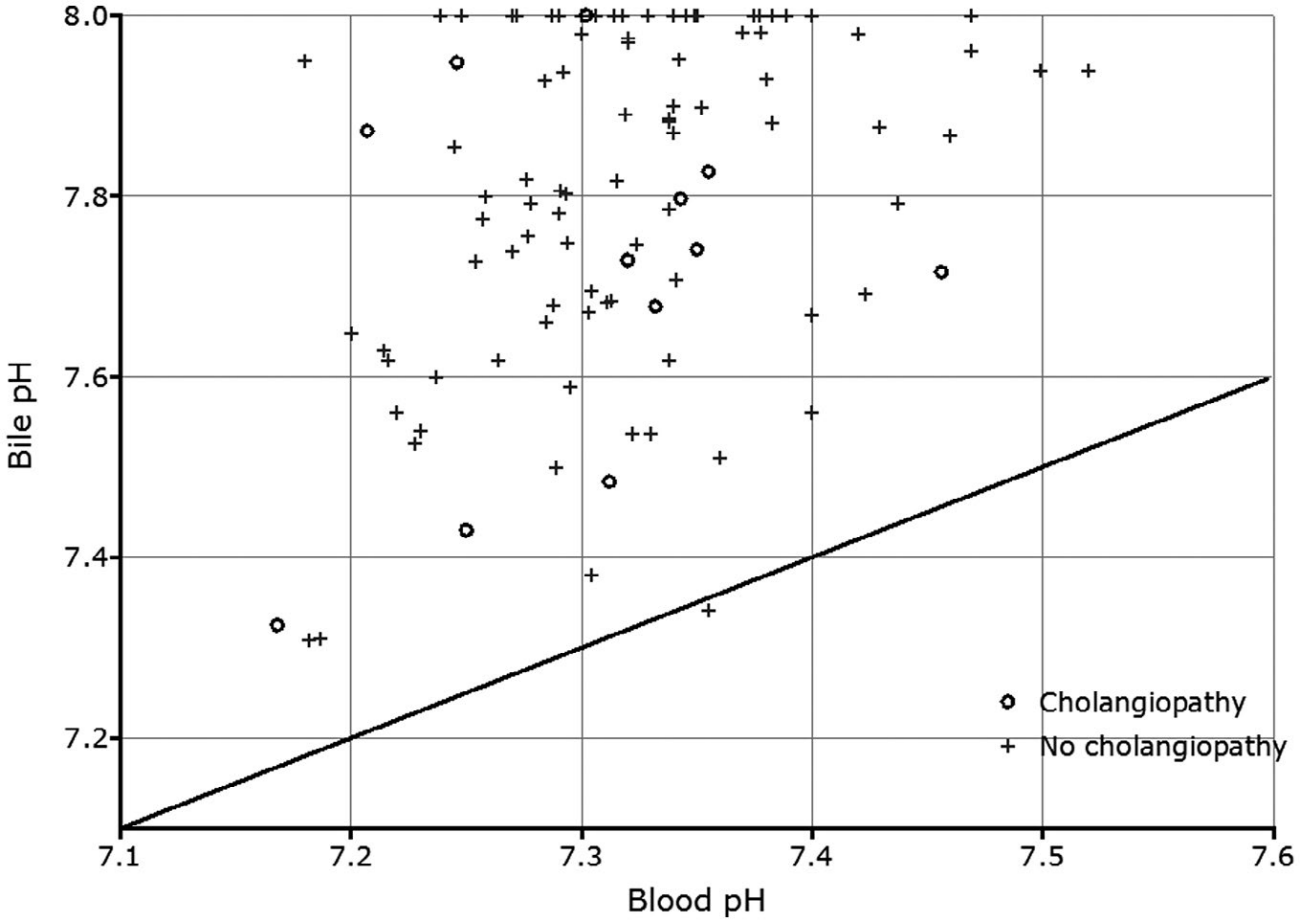
The relationship between bile and blood pH following reperfusion of the liver in the recipients. The straight line represents the equivalence between bile and blood pH. With one exception, bile pH was higher than that of blood. Circles represent the patients with proven cholangiopathy.

**Fig. 3:**
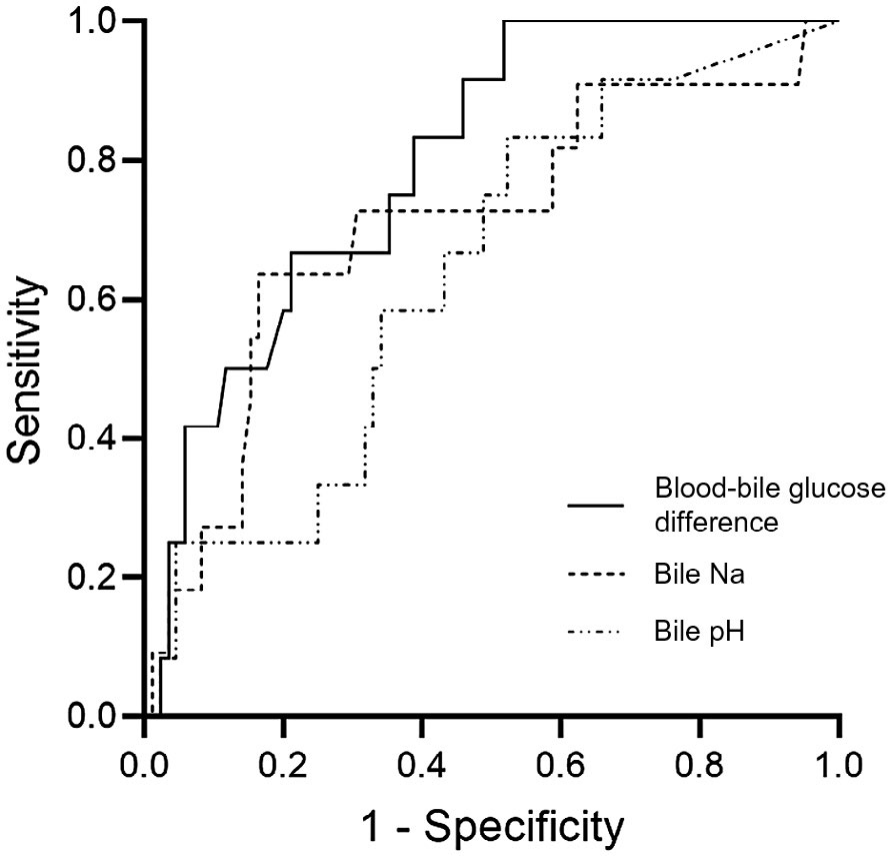
An ROC curve of the blood‐bile glucose difference, bile sodium, and bile pH as a predictor for the development of cholangiopathy. A blood‐bile glucose difference of <6.5 mmol/L has 83% sensitivity and 62% specificity for predicting cholangiopathy (AUROC, 0.80; *P* = 0.001). No such cutoff value can be made from the bile sodium and bile pH. The AUROC (95% CI)s were 0.80 (0.68‐0.91; *P* = 0.001) for the blood‐bile glucose difference; 0.71 (0.53‐0.89; *P* = 0.02) for bile sodium; and 0.64 (0.48‐0.79; *P* = 0.12) for bile pH.

There was no difference between the median time taken for arterial reperfusion between the 12 livers developing and the 88 not developing cholangiopathy (74 versus 80 minutes; *P* = 0.35; Table 1). Similarly, median anastomotic WIT (portal perfusion) was also comparable (37 versus 38 minutes; *P* = 0.17). Median biliary sodium (142 versus 147 mmol/L; *P* = 0.02) and median biliary‐blood sodium difference (3.4 versus 7.7 mmol/L; *P* = 0.01) were significantly lower in the group that developed cholangiopathy (Table 5). Similarly, the median of the difference between glucose concentration in blood and bile was significantly lower in the cholangiopathy group (5.2 versus 7.6 mmol/L; *P* = 0.001). All other bile biochemistry values were similar without any significant difference. Absolute bile pH, bicarbonate, and glucose had no predictive values. Figures 1 and 2 illustrate the relationship of blood‐bile glucose concentration and blood‐bile pH, respectively. An ROC curve (Fig. 3) showed that a blood‐bile glucose difference of <6.5 mmol/L has 83% sensitivity and 62% specificity for predicting cholangiopathy (area under the curve [AUC], 0.80; *P* = 0.001), in a setting where all but 6 of the patients had a blood glucose <8 mmol/L. All patients with cholangiopathy had patent hepatic arteries.

### Magnetic Resonance Grading of Cholangiopathy

All 26 patients who underwent MRCP during follow‐up for clinical reasons were reviewed, and 12 patients had radiological evidence of cholangiopathy. Table 6 shows the relevant bile biochemistry values for these 12 patients. The majority (n = 8, 66.7%) had a moderate degree of cholangiopathy. All but 2 had involvement of peripheral ducts. Five patients had clinically significant cholangiopathy. There were 4 patients who were relisted, 3 of whom were retransplanted. Endoscopic retrograde cholangiopancreatography (ERCP) with dilation and stenting of an anastomotic stricture was performed in 1 recipient. All the others recipients are stable at the time of writing.

**Table 6:** MRI Grading of Cholangiopathy

Patient	Type of Graft	DLI	MRI Grading	Level of Bile Duct Stricture*	Bile pH	Bile Glucose (mmol/L)	Bile‐Blood pH Difference	Blood‐Bile Glucose Difference	Status
1	DBD	1.15	Mild	1	7.73	0.7	0.41	7.9	Alkaline phosphatase resolved
2	DCD	1.49	Mild	C, 1, and P	7.33	2.4	0.16	4.8	Expectant
3	DBD	1.29	Moderate	C, 1, 2, and P	7.43	2.7	0.18	3.0	ERCP plus surgery
4	DCD	1.86	Moderate	1, 2, and P	>8	8.7	0.7	6.4	Expectant
5	DCD	2.02	Moderate	C, 1, 2, and P	7.72	2.2	0.26	3.5	Retransplanted
6	DCD	1.95	Moderate	C, 1, 2, and P	7.95	10	0.7	5.6	Expectant
7	DBD‐NESLiP	0.92	Moderate	C	7.87	1.6	0.67	6.5	Expectant
8	DBD	1.48	Moderate	C, 1, 2, and P	7.48	2.1	0.17	5.7	Retransplanted
9	DCD‐NESLiP	2.04	Moderate	1, 2, and P	7.68	1.7	0.35	7.3	Expectant
10	DCD‐NESLiP	2.68	Moderate	C, 1, 2, and P	7.80	2.9	0.45	2.8	Relisted
11	DCD	3.13	Severe	C, 1, 2, and P	7.74	2.5	0.39	3.7	Died from recurrent cholangiocarcinoma
12	DCD	1.74	Severe	C, 1, 2, and P	7.83	6.6	0.47	2.3	Retransplanted

* C, bile duct confluence; 1, first‐order ducts; 2, second‐order ducts; and P, peripheral ducts.

### Bile Duct Histopathology

Bile duct histopathology was available in 85 patients (DCD 29; DBD 56) among the cohort (Table 7). The degree of damage was staged into 2 groups based on stromal necrosis. Low‐grade bile duct injuries (none to mild) comprised 40 (47.1%) patients, and high‐grade injuries (moderate to severe) included 45 (52.9%). There were 3 (7.5%) patients in the low‐grade injury group and 7 (15.6%) patients in high‐grade injury group who developed cholangiopathy without any significant difference between groups (*P* = 0.32). There was also a lack of correlation between bile gas parameters and the degree of bile duct damage.

**Table 7:** Bile Duct Biopsy and Its Relation With Cholangiopathy

Degree of Damage (Bile Duct Injury)	Overall (n = 85)	No Cholangiopathy (n = 75)	Cholangiopathy (n = 10)	*P* Value
None to mild	40 (47.1)	37 (49.3)	3 (30)	0.32
Moderate to severe	45 (52.9)	38 (50.7)	7 (70)	

NOTE:

Data are given as n (%).

## Discussion

Of the 100 transplanted livers studied, 12 developed cholangiopathy. Both the difference between the glucose concentration measured in the bile and blood and a low bile sodium in the first 30 minutes after reperfusion were predictive of cholangiopathy, although no absolute threshold was noted. The inability to produce an alkaline bile pH in the recipient was not predictive of the future development of cholangiopathy. In addition, we noted no correlation between the histological changes observed in the common bile duct at transplantation and the subsequent development of cholangiopathy.

Cholangiocytes modify bile throughout its course along the biliary tree, adding bicarbonate and removing glucose.^(^ ^8^, ^9^ ^)^ Glucose reabsorption from bile was originally demonstrated in 1974 by Guzelian and Boyer.^(^ ^8^ ^)^ Uptake of glucose from bile has been shown to be the property of sodium‐glucose linked transporter type 1, a Na^+^/glucose cotransporter, whereas another glucose transporter, glucose transporter 1, on the basolateral domain of the cholangiocyte removes glucose. In rats, glucose absorption can be saturated, such that as blood glucose increases above a threshold value, the level of glucose in bile begins to increase, in a similar manner to tubular reabsorption of glucose by the kidney, which also saturates as blood glucose rises, producing glycosuria.^(^ ^8^ ^)^ We have noted a similar phenomenon in livers undergoing ex situ perfusion, and it can also be seen in the livers studied here (Fig. 1). Another factor that may affect the biliary glucose concentration is the rate of bile production: removal of glucose from a large volume of bile flowing past cholangiocytes might exceed the cells’ capacity to absorb it while a small volume may not. Hence, the difference between blood and bile glucose needs to be interpreted with care, particularly at lower blood glucose concentrations when the difference between blood and bile will be low as biliary glucose concentration tends toward 0. Figure 1 illustrates this relationship. We acknowledge that analyzing biliary and blood glucose differences, as we have done here, may be misleading for the reasons just outlined, but we report them to illustrate how cholangiocytes are modifying bile and to show the variability in bile glucose. Both factors highlighted above mean that the ratio of bile glucose to blood glucose may not be an appropriate measure of cholangiocyte function, contrary to the suggestion of other authors.^(^ ^12^, ^13^ ^)^

The biliary secretion of bicarbonate is a protective function of the cholangiocytes against bile acids. An electroneutral sodium‐independent chloride/bicarbonate exchanger (anion exchanger 2) and cyclic adenosine monophosphate–responsive chloride channel (cystic fibrosis transmembrane conductance regulator) have been observed in the apical membrane of these cells.^(^ ^9^, ^10^, ^14^ ^)^ Injury to these channels, as might happen during liver preservation and reperfusion, may lead to the development of cholangiopathies.^(^ ^10^, ^15^ ^)^ In the same way that the capacity to reabsorb glucose can be saturated, one might assume that the ability to produce bicarbonate to deprotonate bile sufficiently might also be subject to saturation. However, we have not seen evidence for this, and there appears to be no similar relation to that seen in respect to glucose (Fig. 2). One observation we have made is that the lack of correlation between pH and bicarbonate concentration raises the possibility that there is another component in bile, in addition to bicarbonate, responsible for deprotonation. However, we have not yet identified this. Instead of a blood‐bile pH difference, an absolute pH < 7.5 was thought to better represent the risk of cholangiopathy from our ex situ perfusion work,^(^ ^4^, ^16^ ^)^ though that has not been shown in this study of bile taken following in situ reperfusion. However, during ex situ perfusion, the pH of bile often started low and rose over the first hour of bile production,^(^ ^4^, ^16^ ^)^ and it may be that if we had taken a bile sample from the second 30 minutes after reperfusion, we would have seen the pH rise in those ducts that did not go on to develop cholangiopathy.

In addition to the caveats regarding bile flow and the ability of cholangiocytes to absorb glucose despite saturation, there are other possible explanations as to why pH and glucose are not definitive markers of irreparable bile duct damage. First, because these are processes that are predominantly of higher‐order ducts, it is unlikely that bile biochemistry will reflect the destruction of cholangiocytes around the common hepatic duct or its main branches, so damage to these ducts will not be predicted. Second, it is possible that ducts in some parts of the liver are damaged while others are well preserved. So, the well‐preserved ducts are able to secrete enough alkali (eg, bicarbonate) to produce alkaline bile, whereas in the presence of damage, glucose may be entering the ducts as part of an inflammatory exudate.

The limitations of this study include problems inherent in the retrospective design. The bile sample was not available in half of the patients over the study time period, and these patients were excluded. Similarly, a bile duct biopsy was not obtained in all the patients for whom a bile sample was available. The numbers in each arm (DBD versus DCD; cholangiopathy versus no cholangiopathy) are small and underpowered to discern subtle group differences. The DCD cohort includes both NESLiP and NRP livers. As a policy at our institute since 2018, DCD livers undergo either of the 2 novel preservation techniques before implantation. This may confound the DCD liver results.

In summary, we described the biochemistry of bile immediately after liver transplantation, and we have shown that the inability to resorb glucose, manifesting either in a high biliary glucose or little difference between bile and blood glucose, and low biliary sodium are associated with subsequent cholangiopathy. We have not been able to define a specific threshold value for pH, glucose, or sodium, but it is possible that finding such a threshold will require more information, such as bile flow rate, to enable interpretation.
